# Structure-Activity Analysis of Gram-positive Bacterium-producing Lasso Peptides with Anti-mycobacterial Activity

**DOI:** 10.1038/srep30375

**Published:** 2016-07-26

**Authors:** Junji Inokoshi, Nobuhiro Koyama, Midori Miyake, Yuji Shimizu, Hiroshi Tomoda

**Affiliations:** 1 Graduate School of Pharmaceutical Sciences, Kitasato University, 5-9-1 Shirokane, Minato-ku, Tokyo 108-8641, Japan

## Abstract

Lariatin A, an 18-residue lasso peptide encoded by the five-gene cluster *larABCDE*, displays potent and selective anti-mycobacterial activity. The structural feature is an *N*-terminal macrolactam ring, through which the *C*-terminal passed to form the rigid lariat-protoknot structure. In the present study, we established a convergent expression system by the strategy in which *larA* mutant gene-carrying plasmids were transformed into *larA*-deficient *Rhodococcus jostii*, and generated 36 lariatin variants of the precursor protein LarA to investigate the biosynthesis and the structure-activity relationships. The mutational analysis revealed that four amino acid residues (Gly1, Arg7, Glu8, and Trp9) in lariatin A are essential for the maturation and production in the biosynthetic machinery. Furthermore, the study on structure-activity relationships demonstrated that Tyr6, Gly11, and Asn14 are responsible for the anti-mycobacterial activity, and the residues at positions 15, 16 and 18 in lariatin A are critical for enhancing the activity. This study will not only provide a useful platform for genetically engineering Gram-positive bacterium-producing lasso peptides, but also an important foundation to rationally design more promising drug candidates for combatting tuberculosis.

Lasso peptides are microbial products ribosomally synthesized and post-translationally modified^1^,^2^. The structural feature of lasso peptides is a macrolactam ring formed by a condensation reaction between the α-amino group of an *N*-terminal amino acid (Cys, Gly or Ser) and the side-chain carboxyl group of Glu or Asp at the positions 7, 8 or 9^2^,^3^. The *C*-terminal tail passes through the ring to form the rigid lariat-protoknot structure. They exhibit a high stability against proteolytic or chemical degradation^4^. Lasso peptides show a variety of biological properties; bacteriocin-like activity against Gram-negative bacteria caused by those produced by Gram-negative bacteria (microcin J25, capistruin and astexins) and promiscuous activities ranging from microbial activity^5^,^6^,^7^,^8^,^9^,^10^,^11^,^12^,^13^,^14^,^15^,^16^ to receptor antagonistic activity^17^,^18^,^19^ and enzyme inhibitory activity^20^,^21^ caused by those produced by Gram-positive bacteria. Lasso peptides were discovered by two different approaches; in the first approach, they were discovered by activity-based screenings, revealing that they happened to have a lasso structure^5^,^11^,^12^, and in the second approach, they were discovered from genome-mining approaches. The number discovered in the second approach has markedly increased for these three years. In particular, the genome-mining strategy was easily accessible for Gram-negative bacteria by transforming potential lasso peptide gene clusters into *Escherichia coli* as a host.

Our group discovered lasso peptides, lariatins A and B, from the activity-based screening^12^. Lariatins show very unique biological activity, namely, antimicrobial activity only active against mycobacteria species including human-pathogenic *Mycobacterium tuberculosis.* Other lasso peptides like microcin J25^5^, capistruin^8^, and siamycins^14^ exhibit selective antimicrobial activity. Recently, lassomycin produced by *Lentzea kentuckyensis* was reported to exhibit selective anti-mycobacterial activity^13^. Lariatins A and B (Fig. 1) consist of 18 or 20 amino acid residues with a macrolactone ring via linkage between Gly1 and Glu8 through which the *C*-terminal tail passes. The threaded tail is entrapped within the ring by the bulky His12 and Asn14. The lariat-protoknot structure of lariatins results in high resistance to denaturation by chaotropic ions and organic solvents. Our group also identified the lariatin biosynthetic gene cluster from the lariatin producing Gram-positive *Rhodococcus jostii* K01-B0171 by analysis of lariatin non-producing *R. jostii* generated by transposon mutagenesis^11^. At that time genome mining approach was not available due to lack of genome information of *R. jostii*. Before lariatin discovery, the genetic determinants for only two lasso peptides produced by Gram-negative bacteria, microcin J25^22^ and capistruin^8^, were firmly established. Capistruin was discovered from the genome mining approach using the biosynthesis gene cluster information of microcin J25. Four genes are needed for the *in vivo* biosynthesis and export of microcin J25 or capistruin. The *mcjA/capA* gene products are matured to lasso peptides by the two enzymes, McjB/CapB and McjC/CapC. The fourth gene products, McjD/CapD, are ABC transporters that pump matured microcin J25 and capistruin out of the cells. The mechanistic gene structure for biosynthesis of lariatins was found to be essentially similar to those from Gram-negative bacteria except for the presence of one extra ORF (*larC* for lariatins), which appears common in the clusters of lasso peptides produced by Gram-positive bacteria^23^. Interestingly, an actinomycetal strain *Streptomonospora alba* was reported to produce a lasso peptide streptomonomicin, whose biosynthetic gene clusters were identified as *stmABCDE*, which was most similar to the biosynthetic gene clusters of lariatins^24^. Subsequent bioinformatics study has shown that LarC, StmE and similar smaller proteins in lasso peptide operons are homologous to the *N*-terminal domain of McjB and other lasso peptide B proteins, while LarD, StmB and likewise proteins are homologous to the *C*-terminal domain of McjB^25^. Therefore, LarC and StmE are considered a part of protease domain. Although the function was unknown, a recent study demonstrated that StmE is involved in the recognition of the precursor peptide StmA^26^.

Although a number of lasso peptides were reported by the genome mining approach, they are mainly produced by proteobcateria, and the example from actinobacteria is limited. Also, few reports have addressed heterologous production of lasso peptides from Gram-positive bacteria. *Streptomyces coelicolor* system was recently reported for the production and the molecular characterization of lasso peptide predicted by MS/MS analysis^27^.

In the present study, we focused on lariatins produced by Gram-positive *R. jostii* K01-B0171 with intriguing anti-mycobacterial activity. Firstly, a convergent expression system to produce lariatin variants was established by the strategy in which *larA* mutant gene-carrying plasmids were transformed into *larA*-deficient *R. jostii* K01-B0171 (Fig. 2A), because heterologous expression strategy using *R. erythropolis* JCM3201 and *R. jostii* JCM11615 as hosts was not successful^23^. Secondly, a library of lariatin variants was generated to investigate the production and anti-mycobacterial activity of the variants. Finally, systematic structure-activity relationships of lariatins were discussed. Our results concluded that four amino acid residues in lariatin A are essential for production and maturation in the biosynthetic machinery and that three amino acid residues of lariatin A are responsible for anti-mycobacterial activity.

## Results

### Establishment of expression system for the production of lariaitn variants

**Figure 2:**
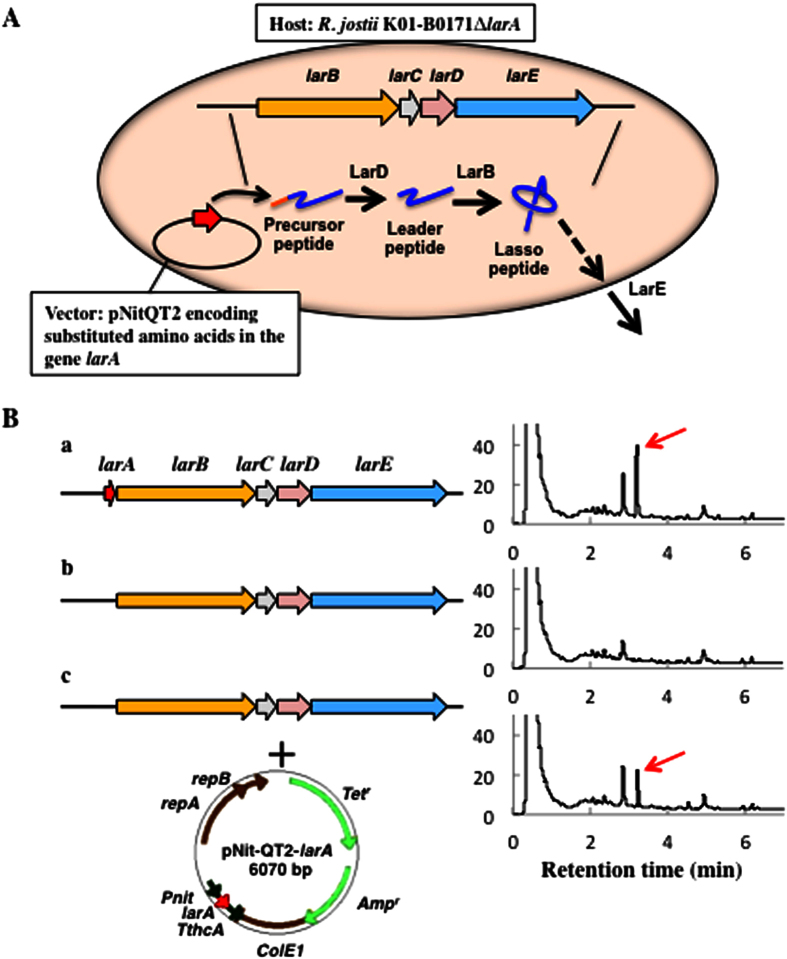
Expression system for the production of lariatin variants. (**A**) Design of the expression system using an actinomycete host. A *larA* deletion mutant was constructed by homologous recombination. The vector that encodes gene sequences for leader peptides was transformed into the mutant carrying *larB-E*, which enabled the expression of various amino acid substituted lariatins. (**B**) Complementation experiments of the *larA* gene. Production of lariatin A was compared among three strains. (a) *R. jostii* K01-B0171 (wild type), (b) *R. jostii* K01-B0171Δ*larA*, and (c) *R. jostii* K01-B0171Δ*larA* + pNit-QT2-*larA.* Red arrow indicates the peak of lariatin A in HPLC data.

Our previous study has shown that a five-gene cluster is responsible for the biosynthesis and export of lariatin A in *R. jostii*; *larA* encodes a precursor peptide consisting of 46 amino acid residues, *larB* and *larD* encode maturation enzymes that convert the precursor peptide into the mature lariatin B, unspecific peptidase are probably relevant to hydrolyze the *C* terminus of lariatin B to yield lariatin A, and *larE* encodes an exporter of lariatin A^23^ (Fig. S1). In this study, we focused on the *larA* deletion mutant from the lariatin-producing bacterium as a host for the production of lariatin variants. To construct the host, the *larA* gene in *R. jostii* K01-B0171 chromosomal DNA was disrupted by insertional inactivation with pk18mob derived from none replication *E. coli* plasmid (pΔlarA). In-frame mutagenesis resulted in the removal of the *larA* gene due to the polar effects of the insertional inactivation on the transcription of genes (Fig. S2). The culture broth of the mutant was analyzed by HPLC and MS. Lariatin A was not detected in the mutant as compared with wild-type strain (Figs 2B and S3). The mutant lacked the ability to produce lariatin A. In contrast, the introduction of pNitQT2*larA* into the mutant restored the production of lariatin A (Fig. 2B). These results indicated that intact *larB* to *larE* in the host genome enabled the biosynthesis and export of lariatin A from the *larA* gene product encoded in the expression vector (Fig. 2A). Thus, the pNitQT2*larA* was utilized as the template for the production of all variants described in this study.

### Analysis of determinants for the maturation and production of lariatin A

**Figure 3:**
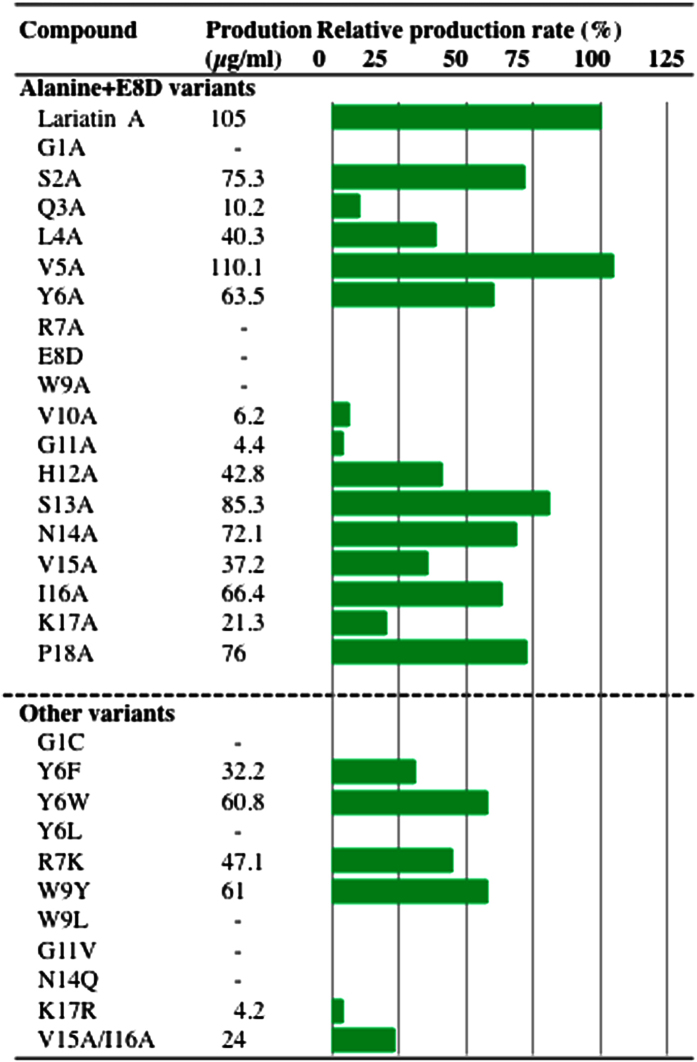
Production of lariatin variants. Supernatant of 5-day culture broth of the strains that genetically expressed each lariatin variant was analyzed by HPLC. After measuring the peak area of each lariatin variant, the producted amounts were estimated based on comparison to the peak area and yield of the lariatin wild type. Minus means that the compounds were not detected in the culture broth. Additionally, green bar represents the relative production rate of each compound when the production of lariatin A is set up at 100%. Value of production amount represents the average from two independent experiments.

The first step in the maturation of lariatin A is the peptide bond cleavage reaction between Ala26 and Gly27 of the precursor protein LarA catalyzed by LarD (Fig. S1). Critical positions of this maturation reaction in the LarA were determined by an alanine scan at the positions −4 to −2 close to the protease cleavage site. When the mutation had no effects on the production of lasso peptides, lariatin A or the related lariatin variants were anticipated to be detectable by HPLC analysis. As a result, the replacement of Arg− 4 and Thr− 3 with alanine didn’t affect the production of lariaitn A, whereas the alanine substitution of Thr− 2 abolished the production of lariatin A (Fig. S4). These results indicated that the residue at position −2 was most closely related to the processing of the precursor LarA by LarD. Next, in order to understand the tolerance of the amino acid sequence within the lasso peptide framework, the production of an E8D variant and an alanine scan in the other positions of the leader peptide sequence were performed. From LC-MS analysis, a doubly protonated ion peak and/or a protonated ion peak expected in the MS spectra was not observed in the four strains that genetically produce G1A, R7A, E8D and W9A variants, although those of the other 14 alanine-substituted variants were detectable (Fig. S5). It was shown that residues within the macrolactam ring except for the positions 1, 7 and 8, and residues at the positions 12 to 18 within the tail part except for the position 9 could be replaceable with alanine without almost effects on the production of lariatins (Fig. 3). Although V10A and G11A variants were detectable, the production amount was much less as compared with other producible variants (Fig. 3).

Furthermore, we designed to genetically produce 11 lariatin variants (G1C, Y6F, Y6W, Y6L, R7K, W9Y, W9L, G11V, N14Q, K17R, and V15A/I16A) for study on the structure-activity relationships as described below. Interestingly, the production analysis provided deeper insights into the maturation and production of lariatin A. As shown in Fig. 3, G1C, Y6L, W9L, G11V, and N14Q were not detectable among the variants. The Gly residue at the position 1 was not tolerant to the substitution of alanine and cysteine, which was consistent with the result of microcin J25 and capistruin^28^,^29^. While the aromatic amino acid residues were favorable at the position 9 because of the loss of the production by the leucine or alanine substitution, the position 6 appears to prefer tryptophan and alanine to tyrosine, and the leucine substitution was not tolerant at the position 6. Similarly, the amino acid residue with a positively charged side chain was favorable at the position7, and the position 11 could not be replaceable with amino acid residues with a bulky side chain. Notably, the Asn at position 14 could not be replaced with Gln, although they have almost identical side chain.

Finally, to investigate the flexibility of the *C* terminus and the macrolactam ring in the lariatin structure, we designed to genetically produce 7 variants related to *C*-terminal truncation, *C*-terminal elongation, ring shortening or ring extension. Insertion of stop codons at Ile16, Lys17 and Pro18 encoded in the *larA* gene was anticipated to yield the lariatin variants of 15, 16 or 17 amino acids in length. However, a protonated ion peak was observed at *m/z* 933 [M + H]^+^ in place of the expected ion in all the strains (Fig. S6). The *m/z* value corresponded to that of the cycloctapeptide composing of Gly1 to Glu8, suggesting that the variants are not processed correctly or unthread after processing and thus form shorter cyclic peptides possibly due to the degradation by unspecific peptidase inside the cells. Furthermore, *C*-terminal elongated 21-residue and 26-residue lariatin variants with one alanine or six histidines were also produced but degraded to yield lariatin A. Also, ring shortening by the removal of Val5, or ring extension by insertion of glycine between Leu4 and Val5 was not successful.

### Anti-mycobacterial activities of lariatin variants

**Figure 4:**
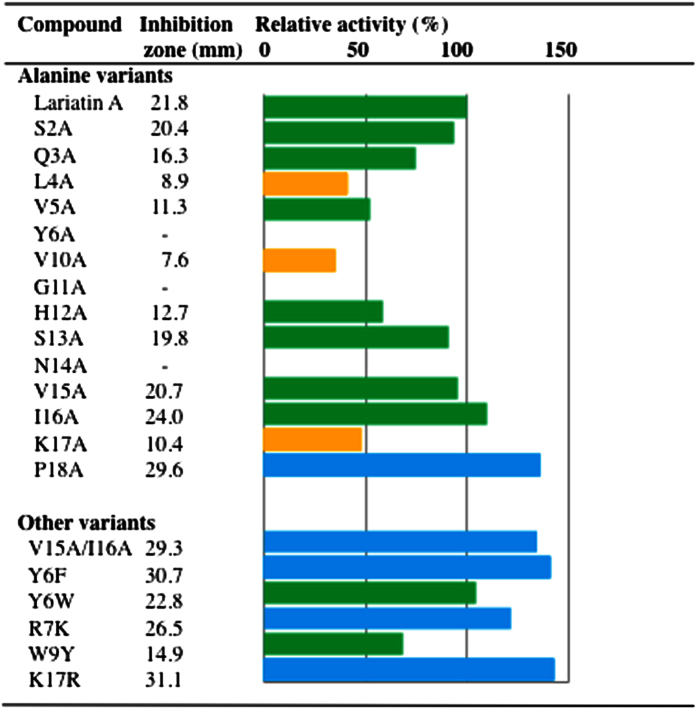
Anti-mycobacterial activity of lariatin variants. Anti-mycobacterial activities of lariatin variants were evaluated by a paper disk method. Value of the inhibition zone against *M. smegmatis* and relative activity in comparison with lariatin A were shown here. Bar color represents potency of each compound when the value of inhibition zone of lariatin A is set up at 100%: yellow, <50% activity; green, 50–120% activity; blue, >120% activity. Value of inhibition zone represents the average from two independent experiments.

To gain insights into the structure-activity relationships of lariatin A, anti-mycobacterial activity of 20 lariatin variants against *M. smegmatis* was evaluated by paper disk method (Fig. 4). Among lariatin variants related to the macrolactam ring, the anti-mycobacterial activity of Tyr6 variant only was lost, while the replacement of Tyr6 with tryptophan and phenylalanine didn’t affect the anti-mycobacterial activity. These results indicated that the aromatic amino acid residue at the residue at position 6 was favorable for the anti-mycobacterial activity. Furthermore, Gly11 and Asn14 were closely involved in the anti-mycobacterial activity because of the loss of the activity in their variants. Also, analysis of Rotating Frame Nuclear Overhauser Effect Spectroscopy (ROESY) indicated that key correlations from His12 to Leu4 and Val5, commonly observed in the lariatins A and B, disappeared in the N14A variant, suggesting the conformational change in the N14A variant (Fig. S7). Regarding the *C* terminus, I16A, K17R, P18A and V15A/I16A variants exhibited more potent activity than lariatin A, while K17A variant had weaker activity. Thus, it was shown that the positively charged amino acid residue was favorable at the residue at position 17. Also, it was shown that the amino acid residue without bulky or branched side chains at the residues at positions 15, 16 and 18 contributed to the higher potency.

## Discussion

In the current study, we established an expression system with *R. jostii* K01-B0171Δ*larA* to enable the robust production of lariatin A, which served as a platform for genetically engineering lariatin variants. The *larA* gene mutagenesis-based approach successfully afforded various lariatin variants by the involvement of the *larB* to *larE* gene products in the maturation and export of lariatins. For lasso peptides from Gram-negative bacteria, homologous fermentation and heterologous expression with *E. coli* were extensively utilized. For lasso peptides from Gram-positive bacteria, heterologous expression system in *S. coelicolor* was reported^27^. In this work, we utilized the *R. jostii* K01-B0171 of the lariatin producer because the two *Rhodococcus* strains were not allowed for the production of lariatin A^23^. To the best of our knowledge, the homologous expression system in *Rhodococcus* sp. was the first report. The *larA*-deficient *R. jostii* system could potentially be applied to heterologously produce other lasso peptides from Gram-positive gene clusters.

The *larA* and the *lar*A mutant genes encode precursor peptides that would mature to lariatin B and variants thereof, whereas the production of lariatin B and the corresponding variants was not confirmed throughout our production experiments. Also, the strains that produce lariatin variants with the additional amino acid at the *C* terminus yielded lariatin A. As the results were observed in other lasso peptides^25^, lariatin B and all the variants also seems to be labile to the hydrolysis by unspecific peptidase in the *C*-terminal region.

The biosynthesis and mutational analysis of lasso peptides such as microcin J25, capistruin, astexin-1, caulosegnin I and xanthomonin II (Fig. S8) were extensively studied. Previous study on the upstream of the leader peptide sequences has shown that threonine residue at the position −2 is indispensable for the maturation step. Actually, such a substitution caused the loss of production of the lariatin variant. The characteristics were very similar to those of microcin J25, capistruin and xanthomonin II, while the production of the corresponding variants in astexin-1 and caulosegnin I was also reported to decrease to the trace amounts. Furthermore, a recent study on caulonodin lasso peptides revealed a new conserved region in the leader peptide sequences of proteobacterial precursor peptides^30^. Interestingly, lariatins also have some of these conserved residues such as the Phe residue at position −6 and the Gly residue at position −8. Other Gram-positive bacteria-producing lasso peptides sviceucin, siamycin I and aborycin similarly contain these residues of Thr-2, Tyr/Phe-6 and Gly-8^4^,^5^,^25^, indicating that these residues in the leader peptide sequences have been conserved cross phylum.

Furthermore, the production analysis of the lariatin variants demonstrated that Gly1, Arg7 Glu8, and Trp9 seem to be needed for maturation of the precursor peptides by the biosynthetic machinery (Figs 3 and S9). The junction of the macrolactam ring of lariatin A could not be replaceable with the similar amino acid Asp having a β-carboxyl group. LarD might be able to recognize the subtle difference between glutamic acid and aspartic acid to make the macrolactam ring by condensation between a α-amino group of glycine at *N* terminal and a γ-carboxylic acid of glutamic acid residue at the position 8. Similarly, replacement studies of microcinJ25 (E8D)^31^, capistruin (D9E)^32^, astexin-1 (D9E)^10^, caulosegnin I (E8D)^33^ and xanthomonin II (E7D)^34^ were reported, but the production of D9E variant in astexin-1 only was confirmed with low production amount. In most of lasso peptides, the junction of the macrolactam rings does not seem to be tolerant to the substitution for similar amino acid residues, suggesting that the enzymes involved in the formation of isopeptide bond of lasso peptides have strict substrate recognition in common. Furthermore, it is reasonable that the production of the G1A, R7A and W9A variants was not detected because the amino acid residues are proximal to the ring junction, and they might interact with the enzyme LarD in the maturation step of lariatin A.

All lariatin variants were produced that contained substitutions of the residues in the β-turn motif (Gly11, His12, Ser13). In previous reports, R11A, V12A and I13A variants in capistruin were not producible^32^, whereas V11A, G12A and I13A variants in microcin J25 were producible^31^. This might be due to the conformational difference of lasso loops. Capistruin and lariatin A have a short lasso loop consisting of 5 amino acid residues, while micorcin J25 has a long lasso loop consisting of 10 amino acid residues. It was suggested that the amino acid substitution that largely changes the conformation of lasso loop might affect the biosynthesis and export of lasso peptides.

Also, the variants whose production was not observed are considered to be inside the cells as the amino acid substitution might affect the transportation by LarE. This is one of interesting questions, but it remains uncharacterized because the cell pellets had higher background. Further study will be needed to define this.

**Figure 5:**
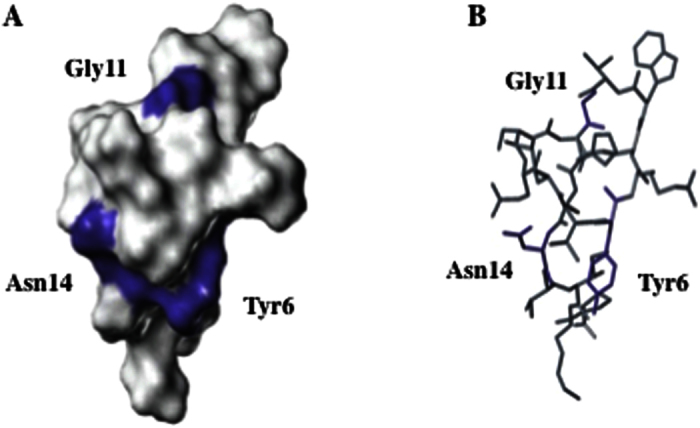
3D structure of lariatin A. Surface model (**A**) and stick model (**B**) are shown here. Purple color shows three amino acid residues (Tyr6, Gly11 and Asn14).

This study on the structure-activity relationship of lariatins demonstrated that Tyr6, Gly11 and Asn14 are responsible for the activity (Figs 4 and S9). Analysis of 3D structure of lariatin A indicated that the Tyr6 is proximal to the Asn14, and interacts each other (Fig. 5). The substitution at the two positions might affect the mutual interaction and cause the loss of the functional structure. It is reasonable that the substitution of Tyr6 and Asn14 significantly affected the activity. Furthermore, lasso peptides generally possess an amino acid residue called “plug” to trap and lock the tail region. Lariatin A also has an amino acid Asn14 corresponding to the plug as supported by the 3D structure and the existence of a bulky chain. As expected, the replacement of the aspartate with an alanine without a bulky chain gave rise to the variant with the distinct conformation from lariatin A (Fig. S7). The reason why the N14A variant lost the activity might be also explainable by this conformational change. Otherwise, the three amino acid residues responsible for the activity might be key residues to interact with the target molecule of lariaitn A in *M. smegmatis*.

Our previous study indicated that the 16-residue variant obtained by the degradation experiment showed much weaker activity than lariatin A^35^. Amino acid length was also important for the anti-mycobacterial activity. Thus, we tried to prepare three variants with 15 to 17 residues, but their products were not obtained possibly due to degradation by peptidase. It was suggested that this part of the tail is needed to maintain a stable lasso fold. This might explain the reason why lariatin A has 18 amino acid residues and no shorter truncations can be observed. More importantly, we found several variants with nearly 1.5-fold increase in potency toward *M. smegmatis*. This suggested that the moderate modification of the peptide structure at the *C*-terminal region has the potential to generate lariatins with greater activity. These findings provide a strategy for preparing a chemical probe that retained the activity to analyze the target molecule.

## Materials and Methods

### Bacterial strains and plasmids

*R. jostii* K01-B0171, the lariatin-producing actinomycete strain^11^, was deposited at International Patent Organism Depositary, National Institute of Advanced Industrial Science and Technology (AIST), Ibaraki, Japan (FERM BP-8267). *E. coli* DH5α was purchased from New England Biolabs for subcloning of plasmid pUC19 and the related plasmids. *M. smegmatis* 607 was purchased from the American type culture collection (Rockville, MD, USA). pNitQT2^26^ and an *E. coli–Rhodococcus* shuttle vector was kindly provided by Dr. T. Tamura (AIST, Ibaraki, Japan).

### Growth and culture conditions

*E. coli* strains were routinely cultured in Luria–Bertani (LB) broth (1.0% Bacto tryptone, 0.5% Bacto yeast extract and 1.0% NaCl), or Luria agar (LA, LB broth containing 1.5% Bacto agar). For lariatin production, *R. jostii* K01-B0171 and its transformants were grown in LP medium (3.0% mannitol, 1.0% glucose, 0.5% yeast extract, 0.5% ammonium succinate, 0.1% KH_2_PO_4_, 0.1% MgSO_4_⋅7H_2_O, 0.0001% FeSO_4_⋅7H_2_O, 0.0001% MgCl_2_⋅4H_2_O, 0.0001% ZnSO_4_⋅7H_2_O, 0.0001% CuSO_4_⋅5H_2_O, and 0.0001% CoCl_2_⋅6H_2_O, pH 7.0 before sterilization). LP agar was made by addition of 1.5% Bacto agar. The antibiotics added into the medium for the selection of transformants were as follows: ampicillin (100 μg/ml for *E. coli*), tetracycline (10 μg/ml and 9.7 μg/ml for solid culture and liquid culture of *R. jostii*, respectively) and kanamycin (30 μg/ml for *E. coli* and 100 μg/ml for *R. jostii*). Optical density at 590 nm was measured to monitor the growth of each bacterium.

### General recombinant DNA techniques

Restriction endonucleases and T4 DNA ligase were purchased from Takara Bio Inc., Shiga, Japan. PCR was carried out using a PCR Thermal Cycler Dice^®^ mini (Takara Bio Inc). QIAprep Spin-Miniprep kit and QIAEX II Gel Extraction kit (Qiagen, Hilden, Germany) were used for DNA isolation from bacterial cells and purification of DNA fragments from agarose gel, respectively. Automatic DNA sequencing was carried out using a BigDye Terminator Cycle Sequencing Ready Reaction Kit and analyzed on an Applied Biosystems 3130 Genetic Analyzer (Applied Biosystems, Carlsbad, CA, USA). Oligonucleotides were purchased from Sigma-Aldrich Japan K.K., Tokyo Japan. The primers used in this study are listed in Table S1. DNA manipulations in *E. coli* were performed according to the method described by Sambrook *et al*.^26^.

### Construction of *larA* deletion mutant of *R. jostii* K01-B0171

For the in-frame deletion of *larA*, the upstream and downstream regions of *larA* were amplified with PCR, and the resulting fragments were cloned into the *Hin*dIII/*Xba*I and *Xba*I/*Eco*RI sites of pK18mob^27^ to give pΔlarA. The upstream and the downstream regions of *larA* was amplified by PCR with KDO–plus DNA polymerase (TOYOBO Co. Osaka, Japan), using pLBC1^23^ as the template DNA, and the primer pairs of larA970Eco and larA2982Xba, and larA3194Xba and larA5659Hin, respectively.

For disruption of the chromosomal genes, the method of in-frame deletion *via* a single crossover with pΔ*larA*. *R. jostii* K01-B0171 was used as described previously^25^. *R. jostii* K01-B0171 transformants were selected by kanamycin (100 μg/ml). The chromosomal DNAs of transformants were amplified with primers larA970Eco and A5659Hin, and the obtained DNA fragment was checked by a restriction map of a DNA fragment. The pΔ*larA*-integrated strains were cultivated in a 500-ml Erlenmeyer flask containing 100 ml LB broth with no antibiotics. After three rounds of cultivation, the kanamycin-sensitive colonies were selected to obtain in-frame deletion mutants designated *R. jostii* K01-B0171Δ*larA*. The genomic structure of the strains obtained was checked by the same method described above and by DNA sequencing using lar337, lar855 and lar1313 primers.

### Construction of *larA* expression vector

The gene *larA* was amplified by PCR with KDO–plus DNA polymerase (TOYOBO Co. Osaka, Japan), primer set of LarA-1FWNde and LarAdPG-STRVHin, and pLBC1 as the template DNA. The fragment was digested with *Nde*I and *Hin*dIII and inserted into pNitQT2 digested with the same enzymes to generate pNitQT2*larA*.

### Construction of expression vectors by site-directed mutagenesis

Lariatin variants were generated by site-directed mutagenesis using PrimeSTAR^®^ Max DNA polymerase (Takara Bio) and pNitQT2*larA* as the template DNA according to the manufacture’s protocol and the integrity of lariatin precursor gene was confirmed by DNA sequencing using primers, QTprimer1 and QTprimer2.

### Production analysis of lariatin A and its variants in a small scale

Each strain was used to inoculate a 500-ml Erlenmeyer flask containing 100 ml LP medium supplemented with 9.3 μg/ml of tetracycline. After incubation at 30 °C for 2 days on a rotary shaker at 200 rpm, the seed culture (5 ml) was transferred into a 500-ml Erlenmeyer flask containing 100 ml LP medium. Fermentation was carried out at 30 °C for 6 days on the same rotary shaker. The whole culture broth was centrifuged at 5000 × g for 15 min. Then, the supernatant was filtered through a 0.45 μm membrane filter for HPLC and LC-MS/MS analysis.

### Analytical conditions by HPLC and LC-MS/MS

For HPLC analysis, Prominence UFLC system (Shimazu Corp., Kyoto, Japan) was used with a diode array detector (SPD-M20A) and a Shin pack XR-ODS (2.2 μm, 2.0 × 50 mm, Shimazu Corp.). For detection of lariatin A and its variants in the culture broth, the samples (culture filtered through a 0.45 μm membrane filter) were analyzed under the following conditions: flow rate, 0.55 ml/min; mobile phase, 6-min linear gradient from 5% CH_3_CN to 40% CH_3_CN containing 0.1% H_3_PO_4_. Under these conditions, lariatin A was eluted with the retention time of 3.1 min. The retention time of other producible variants was shown in each HPLC chromatogram in Fig. S5. The production amount of the variants was calculated based on peak area of isolated variants in this study. The relative production rate of each compound is calculated when the production of lariatin A is set up at 100%.

Lariatin A and its variants were identified based on the result of mass spectrometry. The LC-MS/MS instrument used for this purpose includes Micro mass ZQ (Waters), Separation Module 2795 (Waters) and a diode array detector 2996 (Waters). Lariatin A and variants were separated from other components in the culture broth under the following conditions; column, PEGASIL ODS (2.0 × 50 mm, Senshu Pak); solvent, the 20-min linear gradient from 10% to 100% acetonitrile containing 0.05% trifluoroacetic acid (TFA); column temperature, 40 °C; flow rate, 0.2 ml/min; detection, UV 210 nm.

### Isolation of lariatin variants

Glycerol stock of recombinant clones carrying the corresponding variant production plasmid were used to inoculate a 500-ml Erlenmeyer flask containing 100 ml LP medium supplemented with 9.3 μg/ml tetracycline. The flask was shaken on a rotary shaker at 27 °C for 3 days. The seed culture (100 ml) was transferred into a 7.5l-jar fermenter containing 5 l of the same medium. The fermentation was carried out at 27 °C for 6 days with an aeration of 1.5 l/min. and an agitation of 200 rpm. The culture broth was centrifuged at 5000 × g for 15 min to separate the mycelium and the supernatant. The supernatant was applied onto a column of Diaion HP-20 (60 × 160 mm, Nippon Rensui Co., Tokyo, Japan). After washing with ion exchange water (1.5 l) and 20% acetone (1.5 l), the variants were eluted with 40% and 60% acetone (1.5 l each). The sample was concentrated *in vacuo* to remove the acetone and lyophilized to dryness. The material was dissolved in 50% CH_3_CN and finally purified by HPLC under the following condition; column, a CAPCELL PAK C18 column (20 × 250 mm, Shiseido Co.); solvent, a 30-min linear gradient from 20% to 30% acetonitrile containing 0.05% TFA, flow rate, 8 ml/min; detection, UV 210 nm.

### Assay for the anti-mycobacterial activity

Antimicrobial activity of lariatins against *M. smegmatis* 607 was measured by a paper disk method. The cell suspension of *M. smegmatis* 607 was adjusted to approximately 1.5 × 10^8^ CFU ml^−1^ in a Middlebrook 7H9 broth (Difco, Becton Dickinson, Sparks, MD, USA) containing 0.05% Tween 80 and 10% ADC enrichment (5.0% bovine albumin fraction V, 2.0% dextrose and catalase (30 μg/ml)), and were spread on a Middlebrook 7H9 agar plate. A paper disk (6 ϕ mm, Advantec Toyo Kaisha, Ltd. Tokyo, Japan) containing 1 μg lariatin variants was placed on the agar plate and was incubated at 37 °C for 48 hr. A diameter (mm) of the inhibitory zone observed around the paper disk was measured to evaluate the anti-mycobacterial activity.

### Conformational analysis of the N14A variant

About 10.0 mg of the N14A variant was dissolved in 0.75 ml of D_2_O (Thermo Fisher Scientific K.K. Kanagawa, Japan) for the NMR data acquisition. The measurement of ROESY was carried out according to the condition of lariatin A as reported previously^11^,^35^,^36^,^37^.

## Additional Information

**How to cite this article**: Inokoshi, J. *et al*. Structure-Activity Analysis of Gram-positive Bacterium-producing Lasso Peptides with Anti-mycobacterial Activity. *Sci. Rep.* **6**, 30375; doi: 10.1038/srep30375 (2016).

## Supplementary Material

Supplementary Information

## Figures and Tables

**Figure 1:**
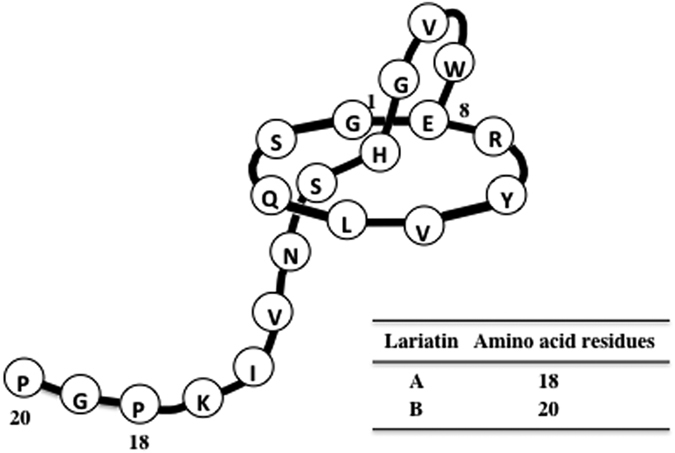
Structures of lariatins A and B.
