# Supplementary material for: Structure-Activity Analysis of Gram-positive Bacterium-producing Lasso Peptides with Anti-mycobacterial Activity
Source: Sci Rep. 2016 Jul 26;6:30375. doi: 10.1038/srep30375 (PMC4960549; doi:10.1038/srep30375)
Supplement: Supplementary Information [file srep30375-s1.pdf]

## Supplemental data

# Structure-Activity Analysis of Gram-positive Bacterium-Producing Lasso Peptides with Anti-mycobacterial Activity

Junji Inokoshi, Nobuhiro Koyama, Midori Miyake, Yuji Shimizu, and Hiroshi Tomoda*

Graduate School of Pharmaceutical Sciences, Kitasato University, 5-9-1 Shirokane, Minato-ku, Tokyo 108-8641, Japan
*Correspondence and requests for materials should be addressed to H. T. (email: tomodah@pharm.kitasato-u.ac.jp)

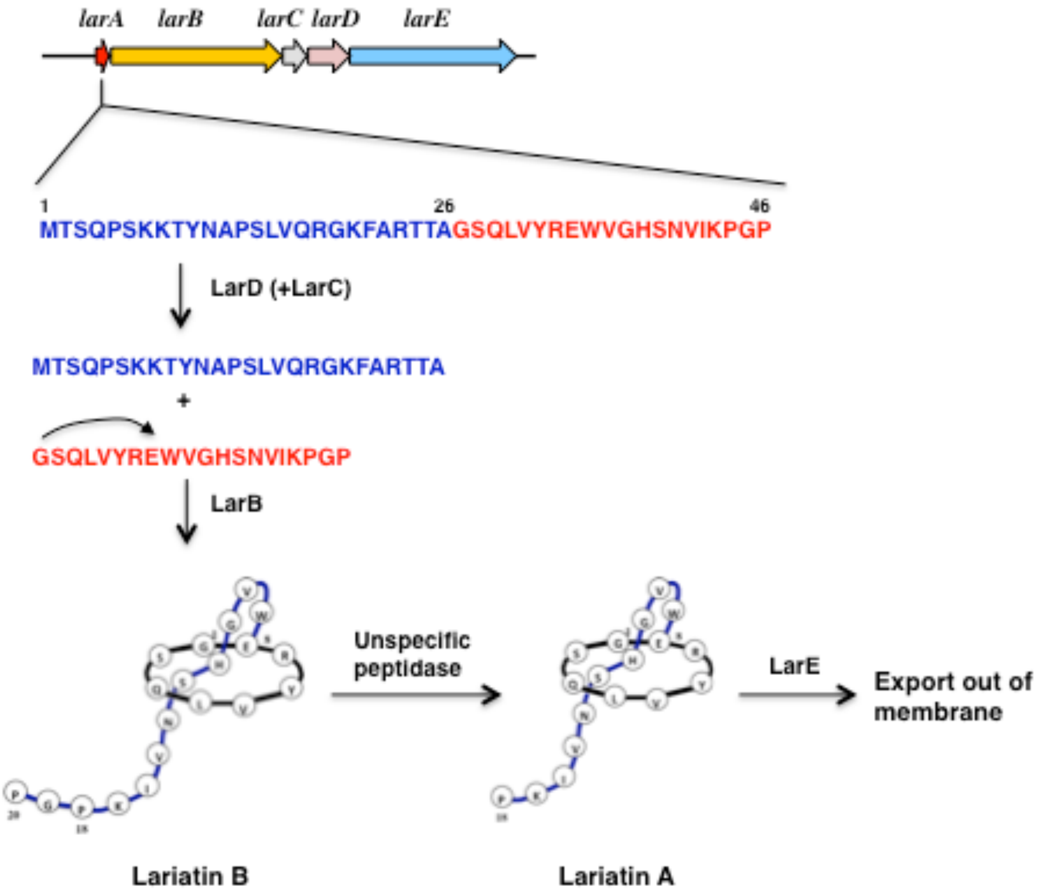


Figure S1 Proposed biosynthesis of the lasso peptide lariatin A.

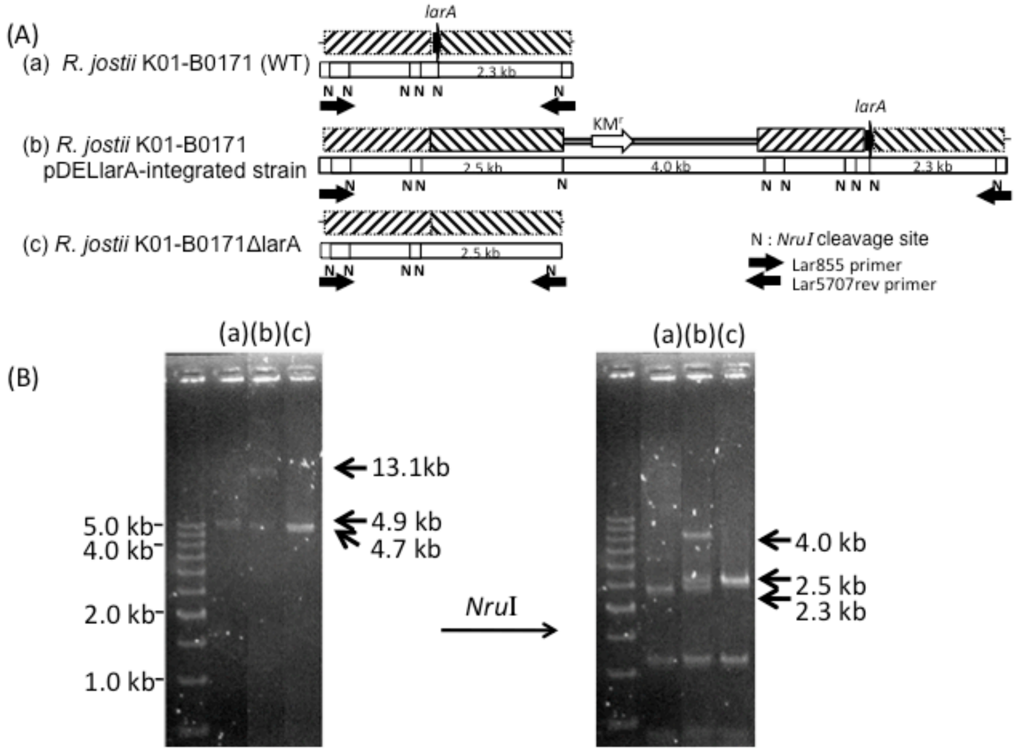


Figure S2 In frame deletion of *larA via* homologous recombination. (A) *Nru* I map of *larA* and its neighborhood region from (a) *R. jostii* K01-B0171 (wild type: WT), (b) *R. jostii* K01-B0171 pDELlarA-integrated strain and (c) *R. jostii* K01-B0171ΔlarA. Each DNA fragment was amplified from chromosomal DNA of the corresponding strains as the template and the primer pairs of lar855 (GCGATTTGTATAGCGGAAGC) and lar5707rev（GATCGTCATGAGGCGAGAGT）. (B) Results of agarose gel electrophoresis of Nru I fragment of the amplified DNA from (a) *R. jostii* K01-B0171 (wild type: WT), (b) *R. jostii* K01-B0171 pDELlarA-integrated strain and (c) *R. jostii* K01-B0171ΔlarA.

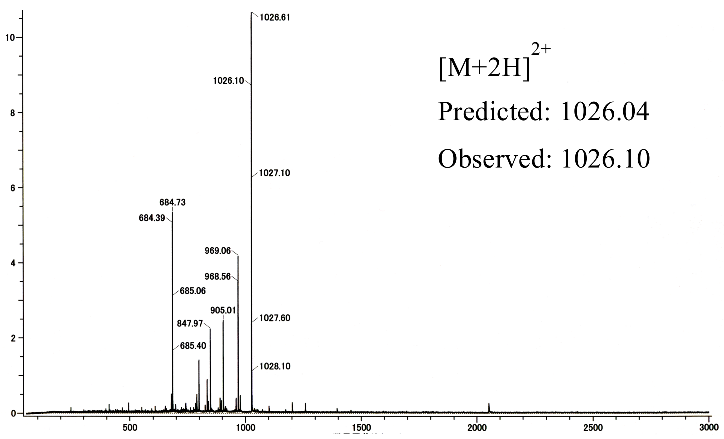


Figure S3 ESI-MS specrum of lariatin A.

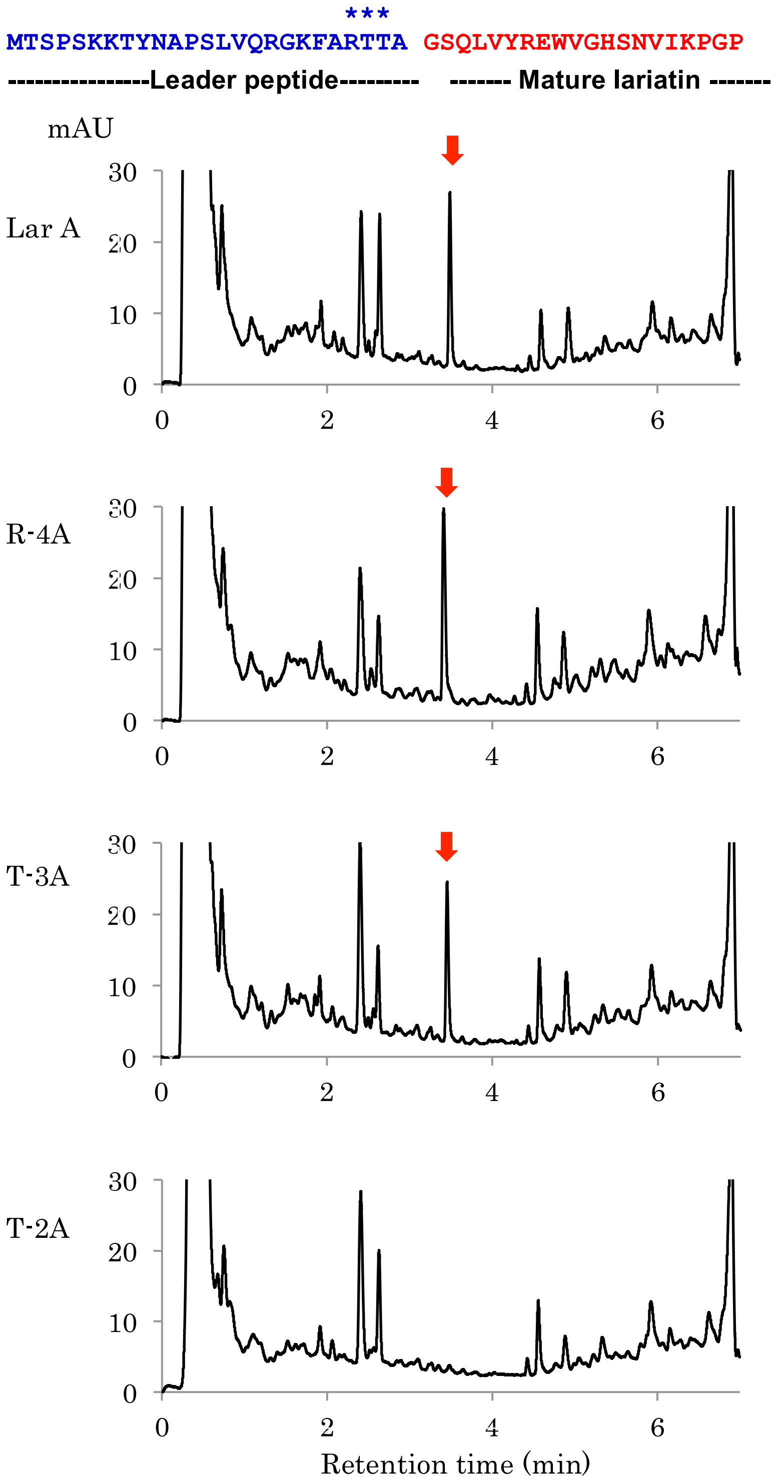


Figure S4 Analysis of lariatin variants (R-4A, T-3A and T-2A) by HPLC. Asterisks show the amino acid residues of the -2 to -4 position at the upstream region in the leader peptide sequence.

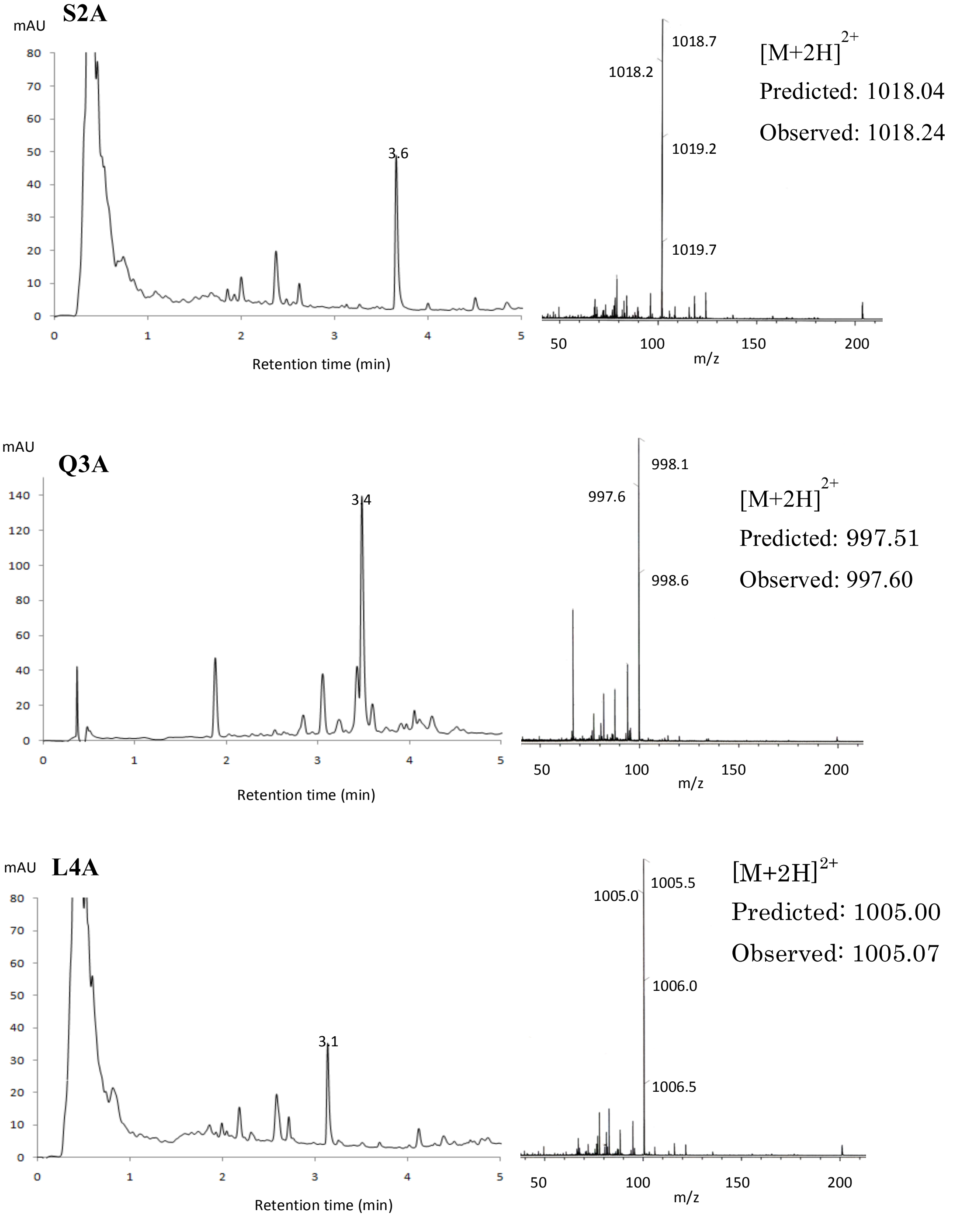


Figure S5 Identification of 20 produced lariatin variants by MS measurements.

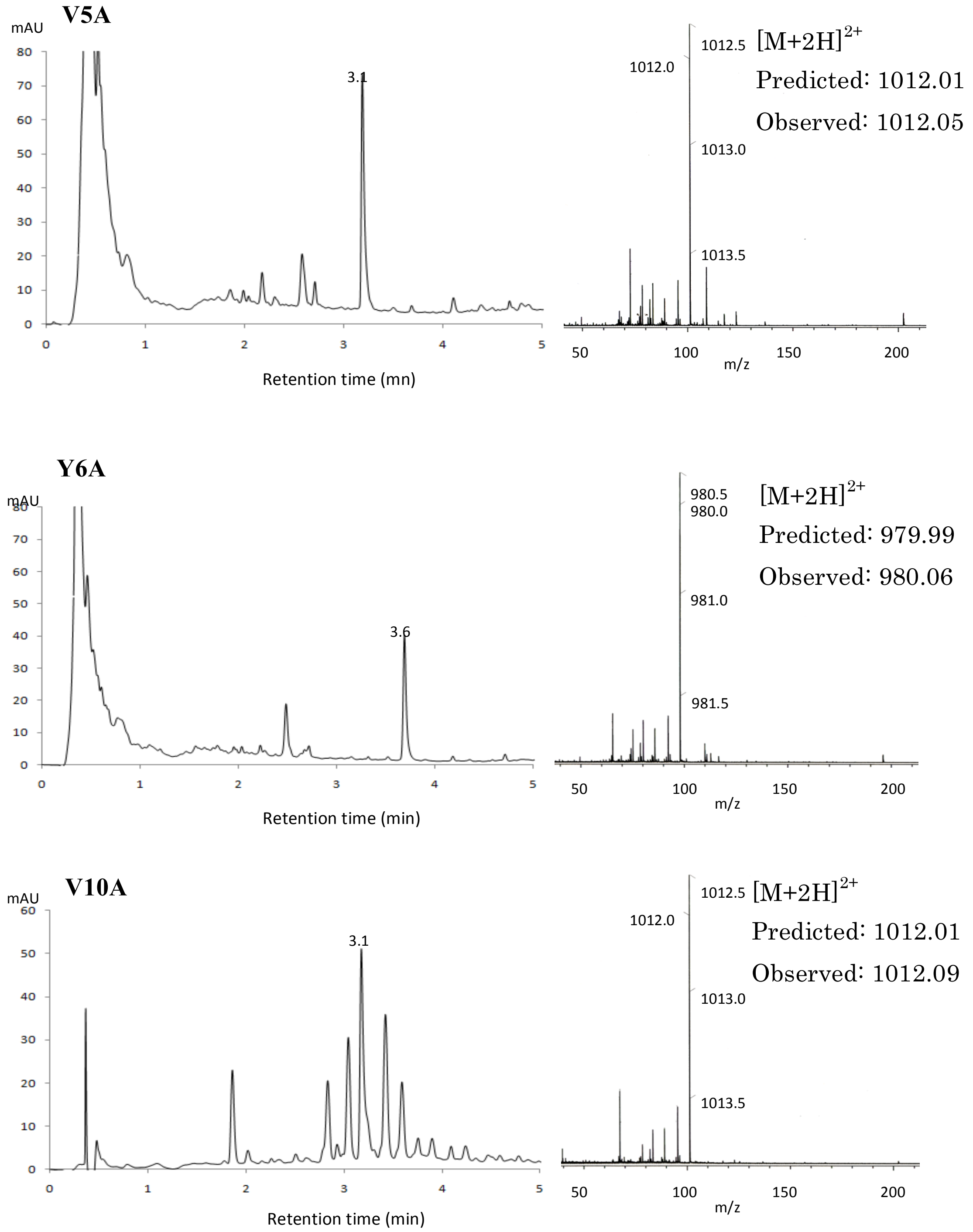


Figure S5 Identification of produced lariatin variants by MS measurements (continued).

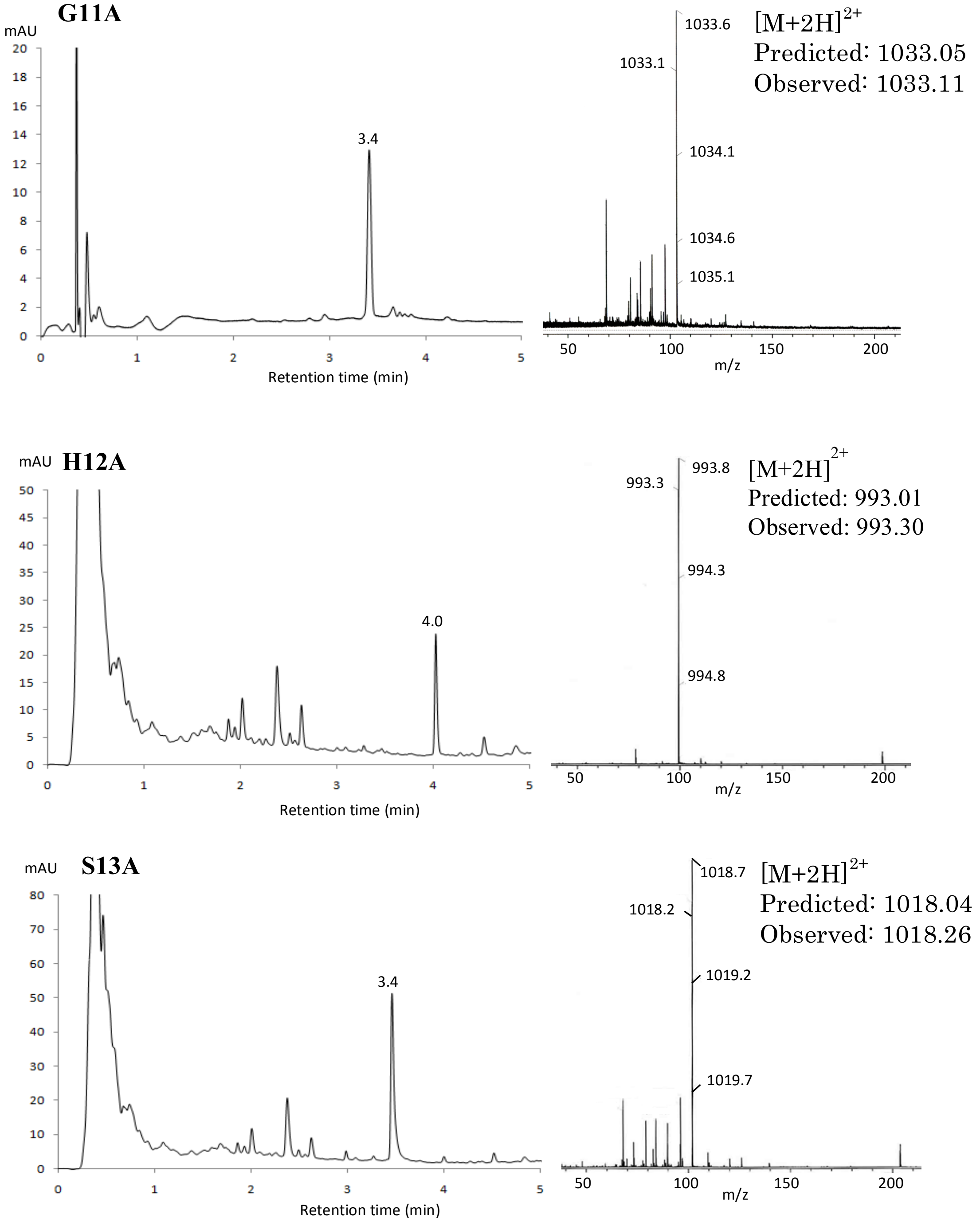


Figure S5 Identification of produced lariatin variants by MS measurements (continued).

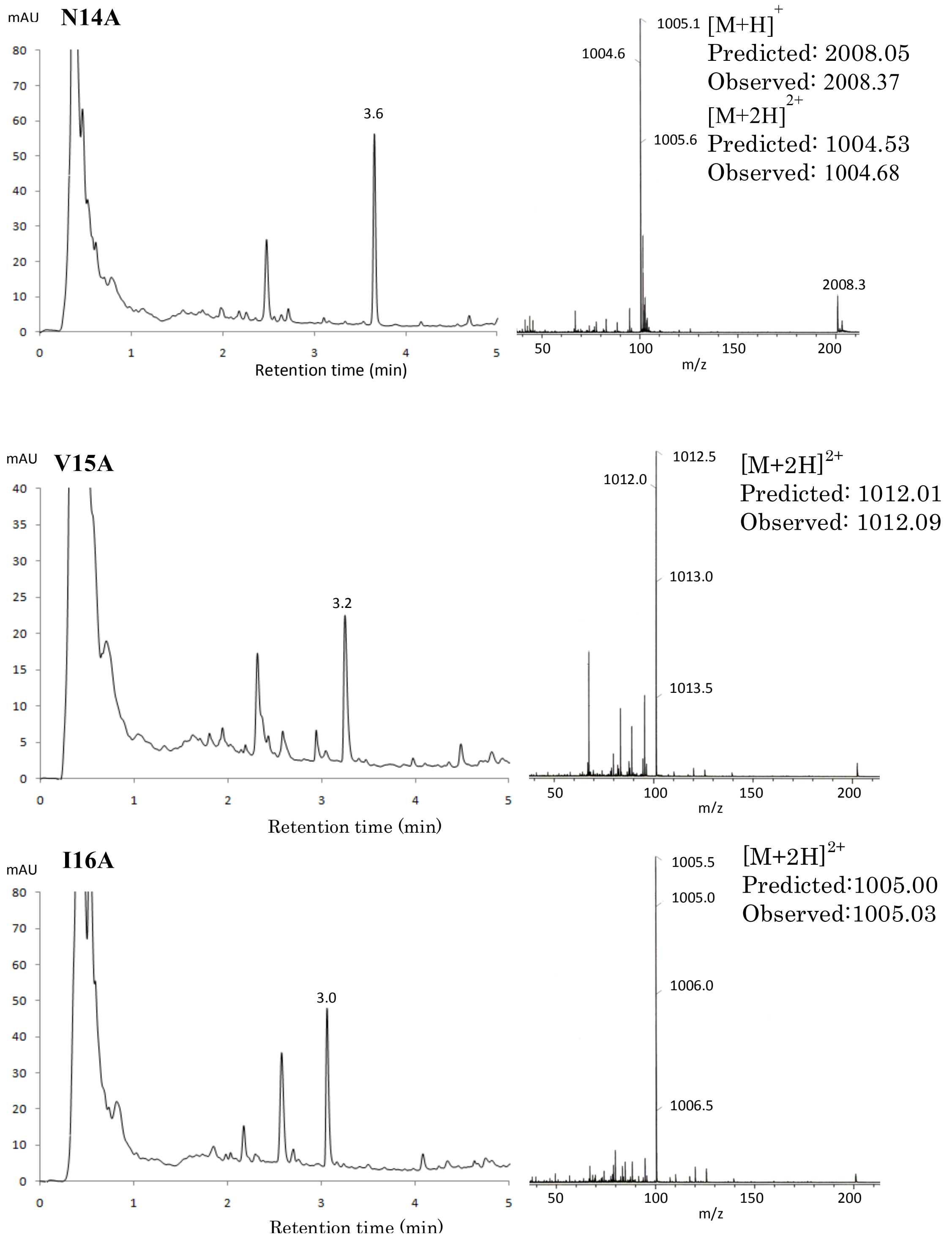


Figure S5 Identification of produced lariatin variants by MS measurements (continued).

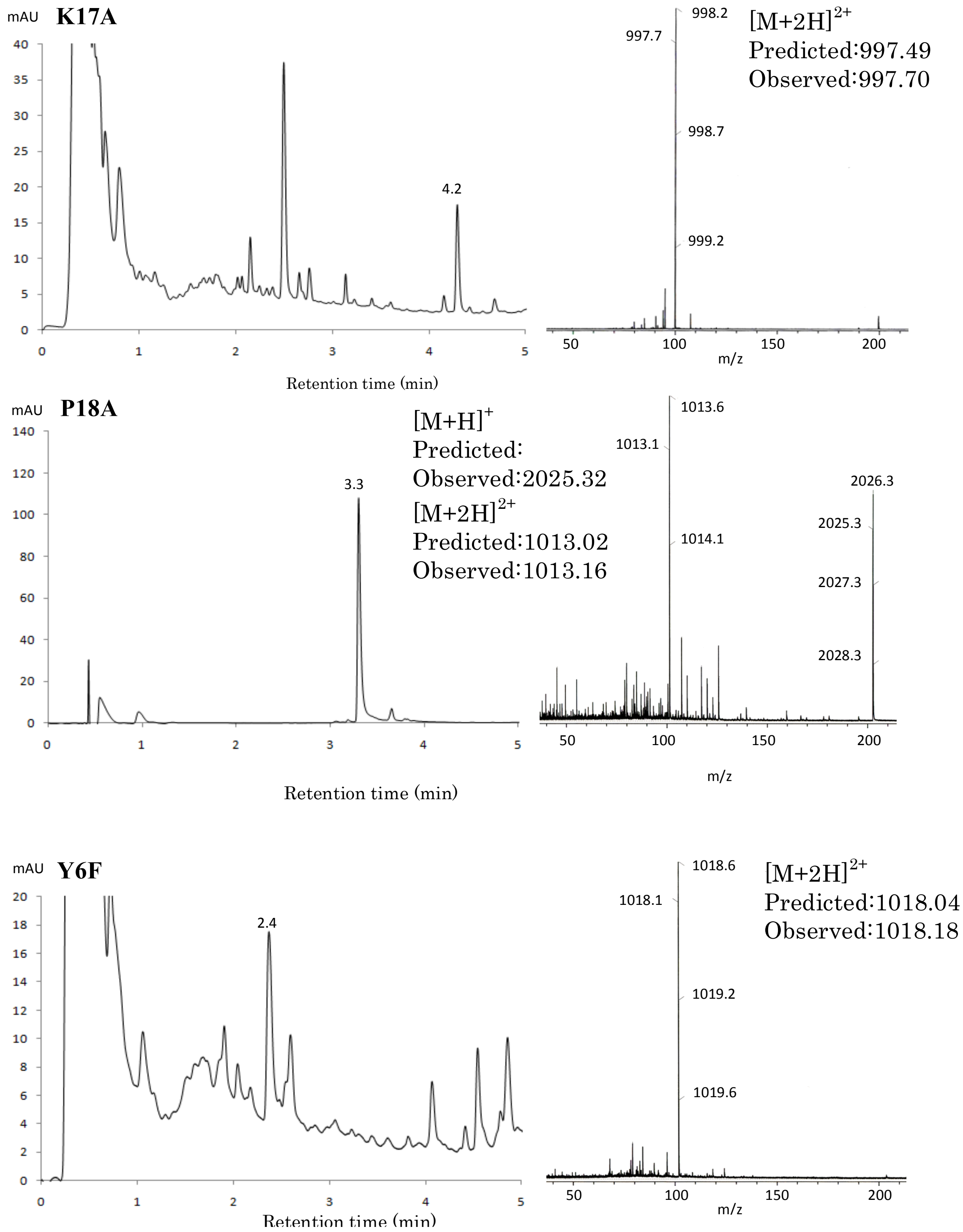


Figure S5 Identification of produced lariatin variants by MS measurements (continued).

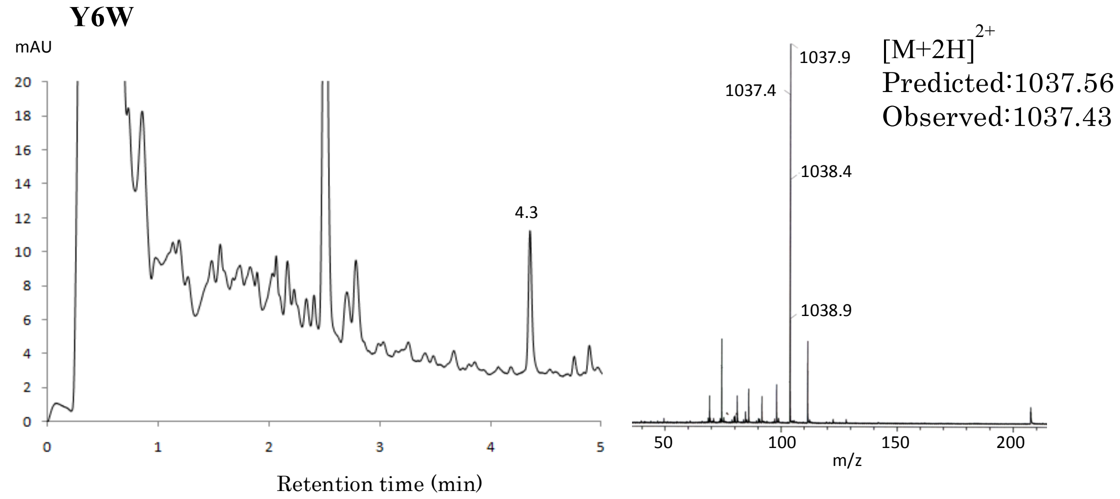


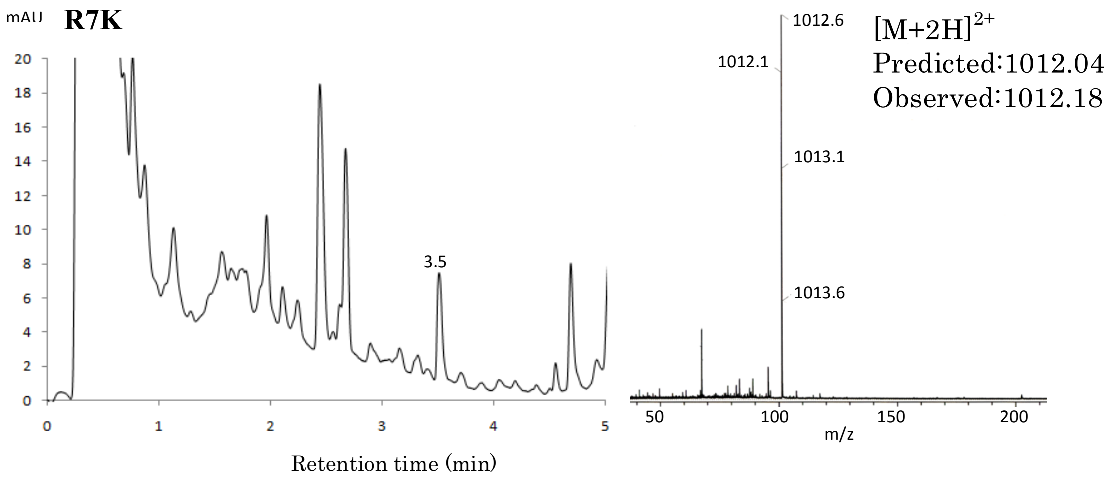


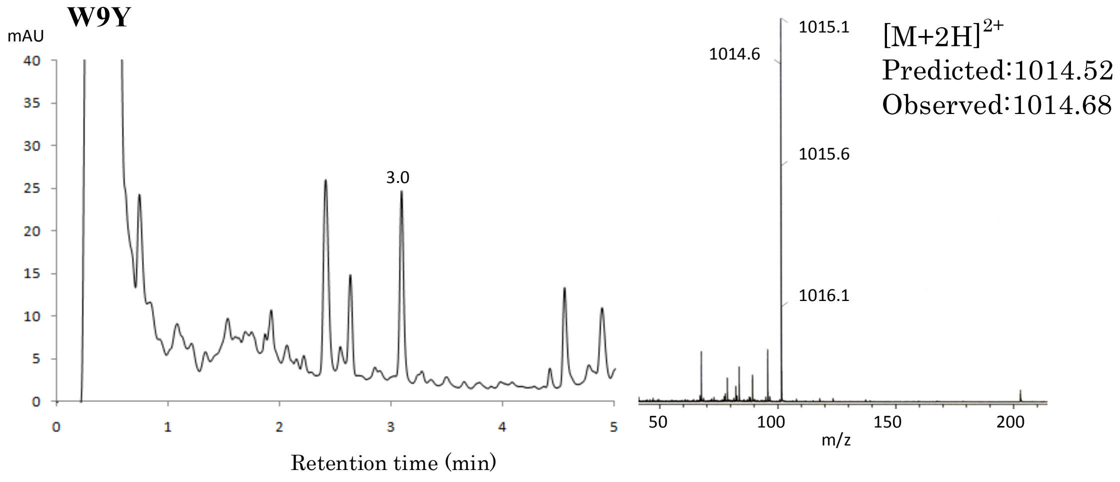


Figure S5 Identification of produced lariatin variants by MS measurements (continued).

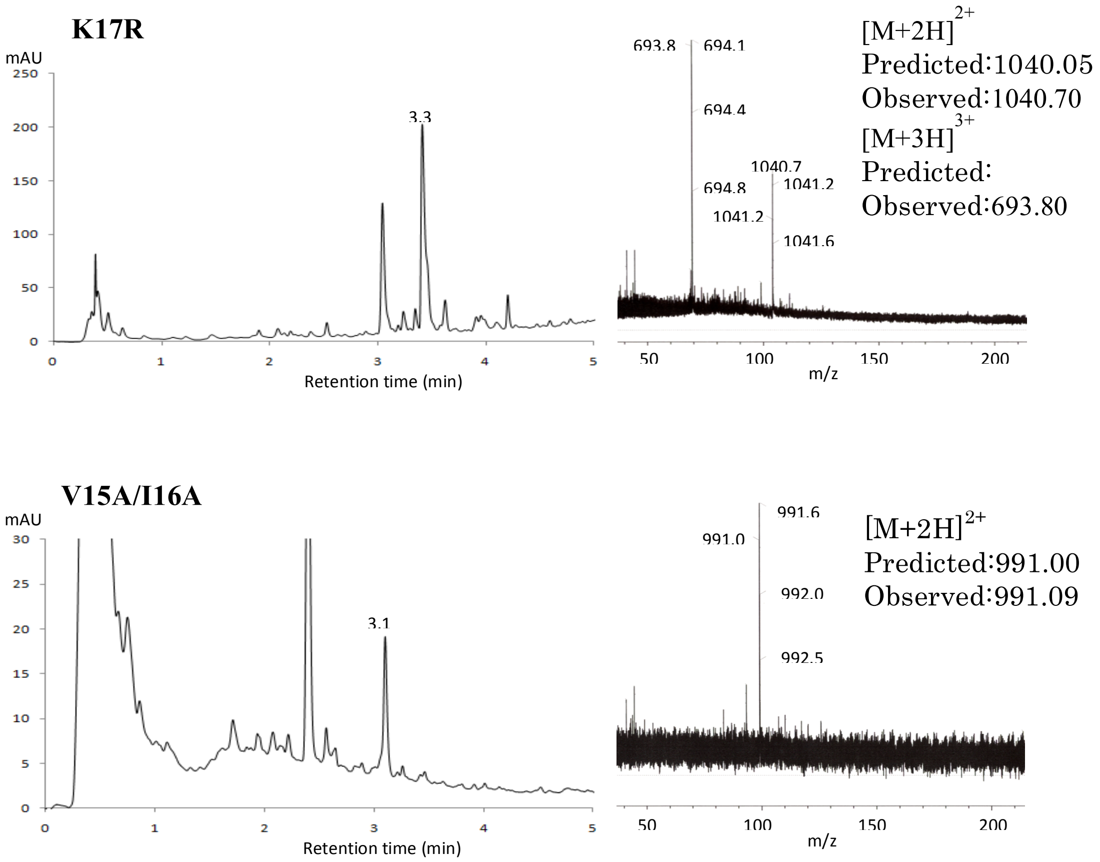


Figure S5 Identification of produced lariatin variants by MS measurements (continued).

**15-residue lariatin variant**

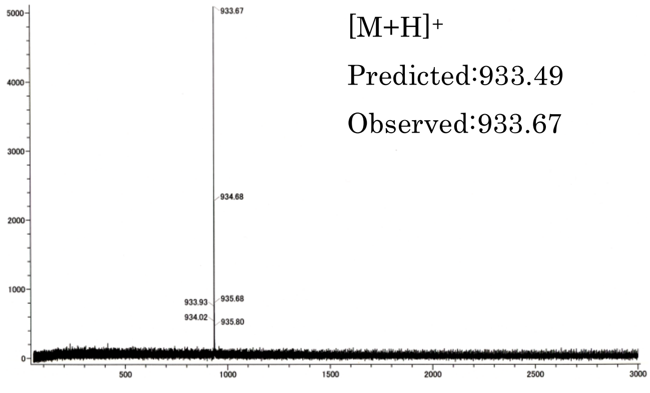


**16-residue lariatin variant**

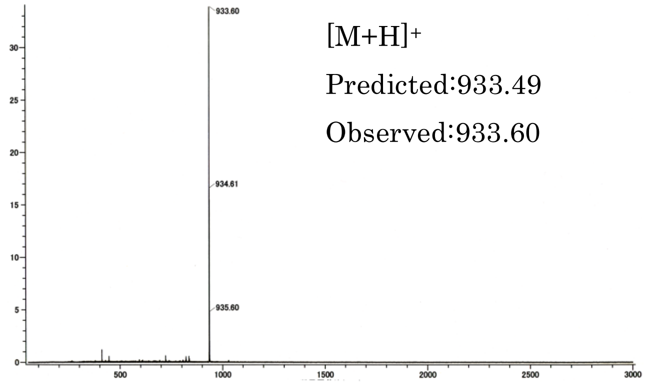


**17-residue lariatin variant**

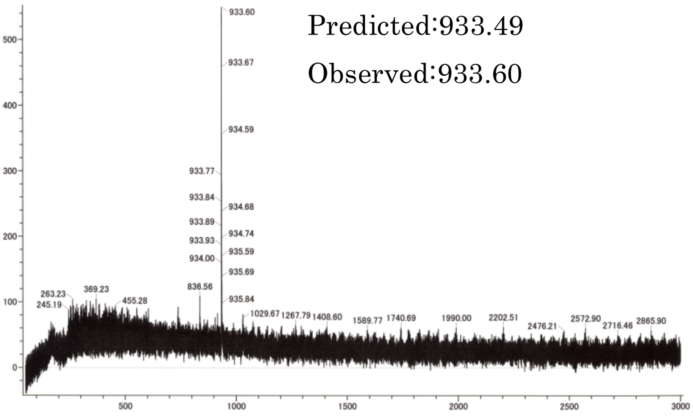


Figure S6 MS spectra of the hydrolyzed product (*m/z* 933 $[M+H]^+$) detected in the 15, 16 and 17-residue lariatin variants.

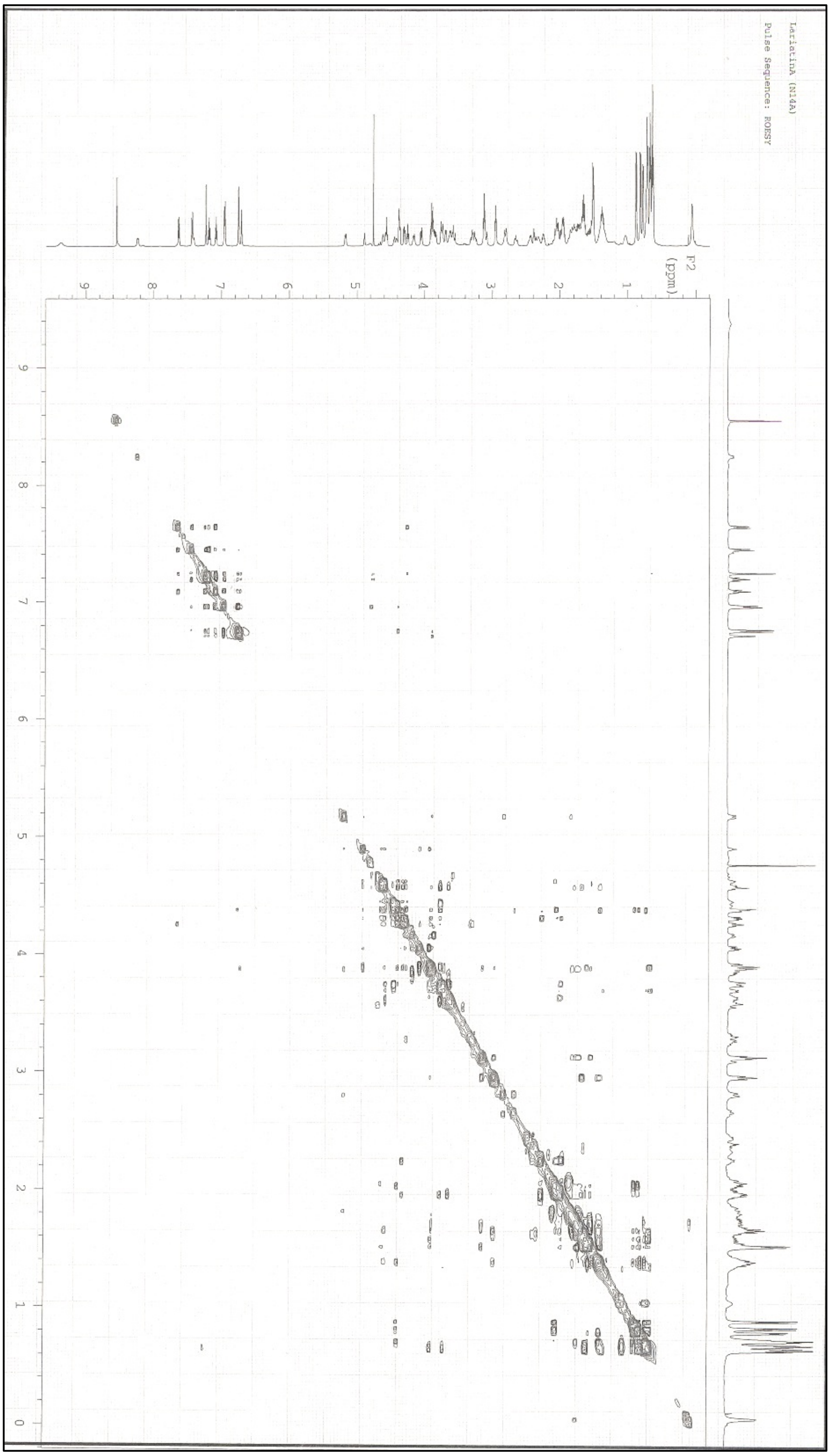


Figure S7 ROESY analysis of the N14A variant in $D_2O$ solution (NMR 400 MHz).

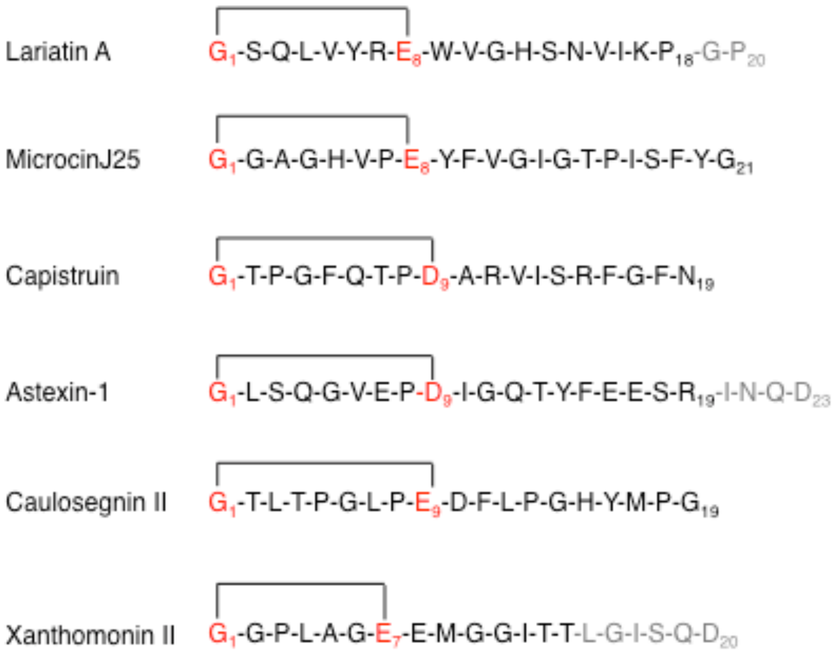


Figure S8 Structures of typical lasso peptides. Red color shows amino acid residues involved in macrolactam ring formation. Gray color shows amino acid residues cleaved off in main product before isolation.

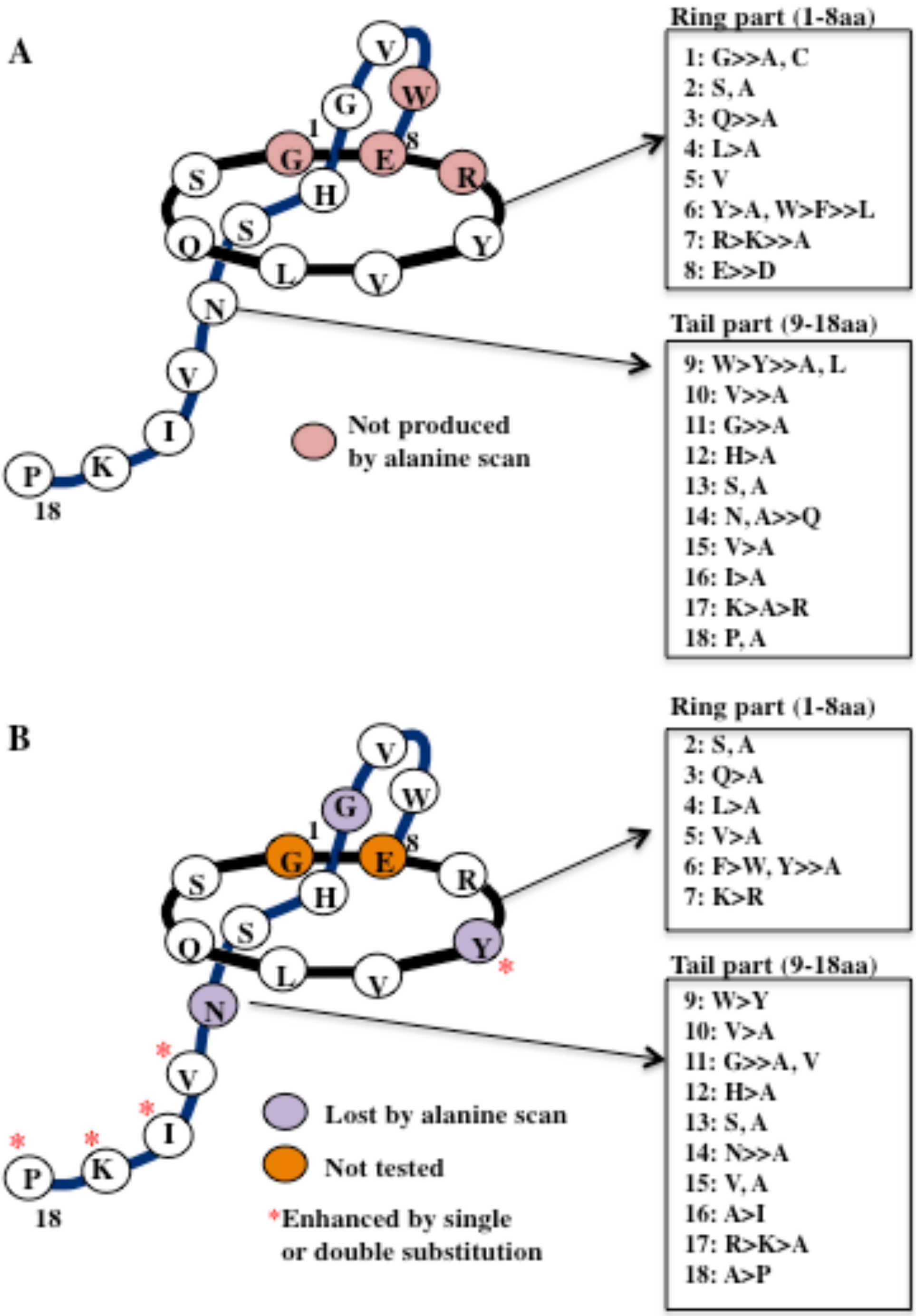


Figure S9 Summary of production and anti-mycobacterial activity of lariatin variants. (A) Relationships between amino acid substitution and productivity. (B) Structure-activity relationships.

Table S1 Oligonucleotide primers used in this study

| Name | Sequence |
|---|---|
| larA970Eco | GGGGAATTCGGCGAAGACCAAAAATGGTA |
| larA2982Xba | GGGTCTAGATTGCGGACAAATTAGCAACA |
| larA3194Xba | GGGTCTAGAGCTCTCTATGTGAAAAGGATGC |
| larA5659Hin | GGGAAGCTTCCTCGATCCAGGCATGTG |
| pTip-larA-Fw | CGGCATATGACCTCTCAGCCGAGCAA |
| pTip-larA-Rv | GCGCTCGAGGGGGCCGGGCTTAATGA |
| PtipPnitQTprimer1 | CCGTGTGTTTGTGCAGGT |
| PtipPnitQTprimer2 | AGGACGCTGGGAGCTTCT |
| Lar-K17A-Fw | GTCATTGCGCCCGGCCCCTAAGCTTA |
| Lar-K17A-Rv | GCCGGGCGCAATGACGTTTGAATGCCC |
| Lar-H12A-Fw | GTCGGGgcTTCAAACGTCATTAAGCC |
| Lar-H12A-Rv | GTTTGAAgcCCCGACCCACTCGCGATA |
| Lar-R7A-Fw | GTCTATgcCGAGTGGGTCGGGCATTC |
| Lar-R7A-Rv | CCACTCGgcATAGACCAGCTGAGAACC |
| Lar-S13A-Fw | CGGGCATgCAAACGTCATTAAGCCCG |
| Lar-S13A-Rv | ACGTTTGcATGCCCGACCCACTCGCG |
| Lar-Y6A-Fw | CTGGTCgcTCGCGAGTGGGTCGGGCA |
| Lar-Y6A-Rv | CTCGCGAgcGACCAGCTGAGAACCTGC |
| Lar-S2A-Fw | AGCAGGTgCTCAGCTGGTCTATCGCG |
| Lar-S2A-Rv | AGCTGAGcACCTGCTGTGGTTCGTGC |
| Lar-E8D-Fw | ATCGCGACTGGGTCGGGCATTCAAAC |
| Lar-E8D-Rv | CGACCCAGTCGCGATAGACCAGCTGA |
| Lar-G1A-Fw | ACAGCAGCTTCTCAGCTGGTCTATCG |
| Lar-G1A-Rv | CTGAGAAGCTGCTGTGGTTCGTGCGA |
| Lar-Q3A-Fw | GGTTCTGCGCTGGTCTATCGCGAGTG |
| Lar-Q3A-Rv | GACCAGCGCAGAACCTGCTGTGGTTCG |
| Lar-L4A-Fw | TCTCAGGCGGTCTATCGCGAGTGGGT |
| Lar-L4A-Rv | ATAGACCGCCTGAGAACCTGCTGTGGT |
| Lar-V5A-Fw | CAGCTGGCCTATCGCGAGTGGGTCGG |
| Lar-V5A-Rv | GCGATAGGCCAGCTGAGAACCTGCTG |
| Lar-W9A-Fw | CGCGAGGCGGTCGGGCATTCAAACGT |
| Lar-W9A-Rv | CCCGACCGCCTCGCGATAGACCAGCTG |
| Lar-V10A-Fw | GAGTGGGCCGGGCATTCAAACGTCAT |
| Lar-V10A-Rv | ATGCCCGGCCCACTCGCGATAGACCA |

| | |
|---|---|
| Lar-G11A-Fw | TGGGTCGCGCATTCAAACGTCATTAA |
| Lar-G11A-Rv | TGAATGCGCGACCCACTCGCGATAGA |
| Lar-N14A-Fw | CATTCAGCCGTCATTAAGCCCGGCCC |
| Lar-N14A-Rv | AATGACGGCTGAATGCCCGACCCACTC |
| Lar-V15A-Fw | TCAAACGCCATTAAGCCCGGCCCCTa |
| Lar-V15A-Rv | CTTAATGGCGTTTGAATGCCCGACCC |
| Lar-I16A-Fw | AACGTCGCTAAGCCCGGCCCCTaagc |
| Lar-I16A-Rv | GGGCTTAGCGACGTTTGAATGCCCGAC |
| Lar-P18A-Fw | CATTAAGGCCGGCCCCTAAGCTTAGA |
| Lar-P18A-Rv | GGGCCGGCCTTAATGACGTTTGAATG |
| Lar-G1C-Fw | ACAGCATGCTCTCAGCTGGTCTATCGC |
| Lar-G1C-Rv | CTGAGAGCATGCTGTGGTTCGTGCGAA |
| T25A-Fw | ACGAACCgcaGCAGGTTCTCAGCTGG |
| T25A-Rv | CCTGCTGcGGTTCGTGCGAACTTCCC |
| T24A-Fw | CGCACGAgccACAGCAGGTTCTCAGC |
| T24A-Rv | GCTGTGGcTCGTGCGAACTTCCCCCG |
| R23A-Fw | GTTCGCAgcaACCACAGCAGGTTCTCA |
| R23A-Rv | TGTGGTTgcTGCGAACTTCCCCCGCTG |
| Y6F-Fw | CTGGTCTtTCGCGAGTGGGTCGGGCAT |
| Y6F-Rv | CTCGCGAaAGACCAGCTGAGAACCTGC |
| R7K-Fw | GTCTATaagGAGTGGGTCGGGCATTCA |
| R7K-Rv | CCACTCcttATAGACCAGCTGAGAACC |
| N14Q-Fw | CATTCAgAaGTCATTAAGCCCGGCCCC |
| N14Q-Rv | AATGACtTcTGAATGCCCGACCCACTC |
| W9Y-Fw | CGCGAGTacGTCGGGCATTCAAACGTC |
| W9Y-Rv | CCCGACgtACTCGCGATAGACCAGCT |
| L4-G-V5-Fw | CAGCTGggtGTCTATCGCGAGTGGGTC |
| L4-G-V5-Rv | ATAGACaccCAGCTGAGAACCTGCTGT |
| L4-A-V5-Fw | CAGCTGgcaGTCTATCGCGAGTGGGTC |
| L4-A-V5-Rv | ATAGACtgcCAGCTGAGAACCTGCTGT |
| V5del-Fw | TCAGCTGTATCGCGAGTGGGTCGGG |
| V5del-Rv | TCGCGATACAGCTGAGAACCTGCTGT |
| Lar-K17R-Fw | GTCATTAgGCCCGGCCCCTaagcttag |
| Lar-K17R-Rv | GCCGGGCcTAATGACGTTTGAATGCCC |
| dGP-V15A/I16A-Fw | GcCgcTAAGCCCTaagcttagatctctcga |
| dGP-V15A/I16A-Rv | AGGGCTTAgcGgCGTTTGAATGCCCGACCC |

| | |
|---|---|
| P18A/His | GCGCTCGAGGGcCTTAATGACGTTTG |
